# EssC: domain structures inform on the elusive translocation channel in the Type VII secretion system

**DOI:** 10.1042/BCJ20160257

**Published:** 2016-06-28

**Authors:** Martin Zoltner, Wui M.A.V. Ng, Jillian J. Money, Paul K. Fyfe, Holger Kneuper, Tracy Palmer, William N. Hunter

**Affiliations:** * School of Life Sciences, University of Dundee, Dow Street, Dundee DD1 5EH, U.K.

**Keywords:** ATPase, ESX-1, forkhead-associated domain, membrane-bound protein, P-loop-containing domain, secretion system

## Abstract

The membrane-bound protein EssC is an integral component of the bacterial Type VII secretion system (T7SS), which is a determinant of virulence in important Gram-positive pathogens. The protein is predicted to consist of an intracellular repeat of forkhead-associated (FHA) domains at the N-terminus, two transmembrane helices and three P-loop-containing ATPase-type domains, D1–D3, forming the C-terminal intracellular segment. We present crystal structures of the N-terminal FHA domains (EssC-N) and a C-terminal fragment EssC-C from *Geobacillus thermodenitrificans*, encompassing two of the ATPase-type modules, D2 and D3. Module D2 binds ATP with high affinity whereas D3 does not. The EssC-N and EssC-C constructs are monomeric in solution, but the full-length recombinant protein, with a molecular mass of approximately 169 kDa, forms a multimer of approximately 1 MDa. The observation of protomer contacts in the crystal structure of EssC-C together with similarity to the DNA translocase FtsK, suggests a model for a hexameric EssC assembly. Such an observation potentially identifies the key, and to date elusive, component of pore formation required for secretion by this recently discovered secretion system. The juxtaposition of the FHA domains suggests potential for interacting with other components of the secretion system. The structural data were used to guide an analysis of which domains are required for the T7SS machine to function in pathogenic *Staphylococcus aureus*. The extreme C-terminal ATPase domain appears to be essential for EssC activity as a key part of the T7SS, whereas D2 and FHA domains are required for the production of a stable and functional protein.

## Abstract (precis)

Structural dissection of EssC, a membrane-bound component of the bacterial Type VII secretion system, reveals two β-sandwich domains at the N-terminus and two ATPase domains at the C-terminus. A structure for the potential pore of the secretion system is proposed.

## INTRODUCTION

The specialized Type VII secretion system (T7SS) is employed by many species of Gram-positive bacteria to translocate proteins across the cell envelope. In the important pathogens *Mycobacterium tuberculosis* and *Staphylococcus aureus* this complex secretion apparatus is a major determinant of virulence [1,2]. The composition of the T7SS and nature of secreted substrates is surprisingly diverse across phyla [3]. However, a characteristic of this secretion system is the presence of two types of protein, the ESAT-6 (early secreted antigenic target of 6 kDA)/EsxA family of virulence factors, which represent the prototypical substrate, and a large integral membrane-bound protein predicted to possess multiple ATPase-type domains at the C-terminal region. These domains are related to the ATP hydrolase DNA translocase FtsK (filamenting temperature-sensitive mutant K) first identified in *Escherichia coli* [4] and the stage III sporulation protein from *Bacillus subtilis* (called SpoIIIE [5]). FtsK and SpoIIIE form oligomeric assemblies with an active site created by subunit pairing where ATP hydrolysis is utilized to induce conformational changes that drive molecular transport. Extending from the conservation of EssC across species there are additional strands of evidence to support a central role for this type of ATPase in the T7SS. The FtsK/SpoIIIE orthologue EccC_5_ is essential to form a stable T7SS membrane complex in *M. tuberculosis* [6]. Furthermore, the homologous EssC in *S. aureus* is required for the secretion of ESAT-6 proteins and EssC-knockout mutants lose the ability to establish persistent infections [2,3].

**Figure 1:**
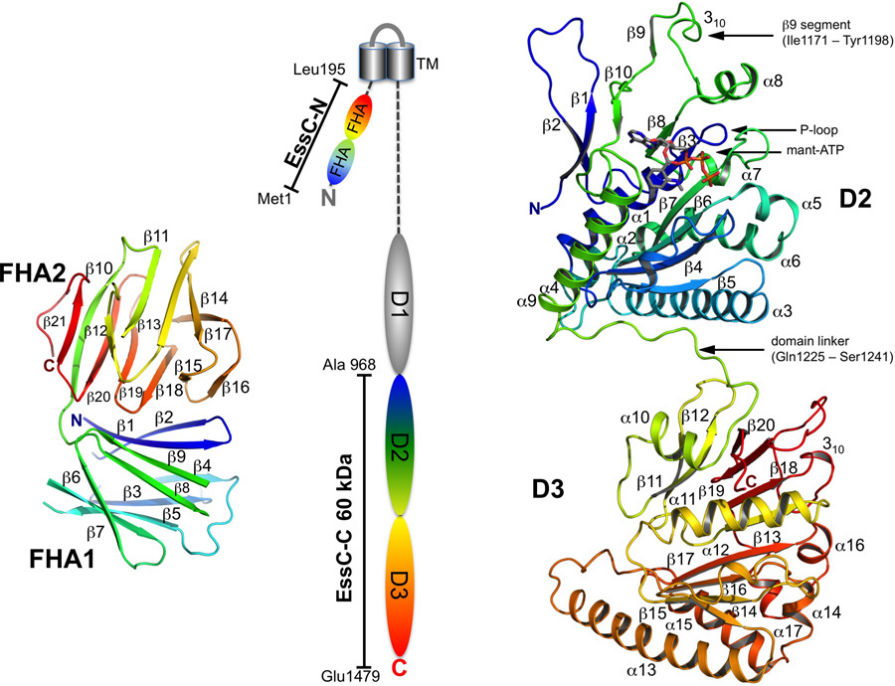
EssC topology and EssC-C structure Cartoon representation of EssC-N (left) in rainbow colour gradient (N-terminus blue to C-terminus red), consisting of two FHA domains. In the topology cartoon of full-length EssC (middle) the structurally characterized domains are drawn in the same colour scheme. Two transmembrane segments (grey cylinders) are predicted with the N-terminal 235 residue peptide, including EssC-N, in the cytoplasm. Cartoon representation of EssC-C (right) in rainbow colour gradient (N-terminus blue to C-terminus red). The bound nucleotide analogue mant-ATP is drawn as sticks. The loop connecting the two ATPase domains, the bound nucleotide, the P-loop and the swapped β9-segment feature are identified.

To investigate EssC, the key core component of this important bacterial secretion system, we targeted the protein from the thermophilic bacterium *Geobacillus thermodenitrificans*. The full-length membrane-bound EssC was heterologously expressed in *E. coli* and the purified detergent-solubilized protein formed an oligomer. Bioinformatics analysis predicts two cytoplasmic components for this 169 kDa integral membrane protein (Figure 1). An N-terminal 26 kDa fragment (EssC-N) composed of two forkhead-associated (FHA) domains, similar to the *S. aureus* orthologue [7], is followed by two transmembrane helices. The C-terminal segment carries three predicted ATPase-like modules or domains, termed D1, D2 and D3. The crystal structures of EssC-N and EssC-C, a C-terminal fragment comprising D2 and D3 (EssC-C, approximately 60 kDa), were determined exploiting single-wavelength anomalous diffraction (SAD) methods applied to selenomethionine (SeMet) derivatives. Analysis of the molecular packing in the crystal lattice of EssC-C and structural comparisons with FtsK suggests a model for the translocation channel, which has so far evaded definitive identification.

While the present study was in progress, Rosenberg et al. [8] published an analysis of the ATPase domains of the EssC orthologue EccC, with an emphasis on the *M. tuberculosis* system. In common with our approach, they included proteins from thermophilic bacteria for analyses and this allows us to compare structures of similar EssC-C constructs of the D2–D3 combination. The comparison reveals a major conformational difference involving D2 that might be linked to the function of the transport system, and a minor perturbation in D3 that correlates with a difference in ATP binding. Rosenberg et al. [8] proposed that the ATPase domain D1 was auto inhibited by the adjacent D2 domain and extended their analyses to include yeast two-hybrid binding studies that indicated binding of the ESAT-6 protein EsxB to EccC and, as will be discussed, a model of oligomerization dependent on complex formation with EsxB. Informed by our structural data on the *G. thermodenitrificans* protein, we investigated the contributions that specific domains of EssC might make to the T7SS of the related organism *S. aureus*.

## MATERIALS AND METHODS

### Cloning

The full-length gene for *G. thermodenitrificans* NG80-2 EssC [Uniprot: A4IKE7, codon optimized for *E. coli* K12 (Genscript)] was cloned into the NcoI/XhoI site of a modified pET27b vector (Novagen) creating pET27bEssC. A construct to produce the N-terminal EssC-N fragment (residues 1–196) was PCR amplified from the full-length gene and cloned into the NcoI/XhoI site of a modified pET27b vector (Novagen) creating pET27b EssC-N. A construct to produce the C-terminal fragment EssC-C (residues 966–1479) was cloned into the same vector. Both plasmids produce an N-terminal hexahistidine-tagged protein with a tobacco etch virus (TEV) protease cleavage site.

Full-length *S. aureus essC* (Uniprot: P0CO48) and the various domain-deletion constructs (FHA, D23 and D3) were amplified from genomic DNA (strain RN6390) and cloned into the BglII/EcoRI sites of plasmid pRMC2 [9]. Haemagglutinin (HA)-tagged variants of full-length *ess*C and domain deletion constructs D23 and D3 were amplified from the respective untagged constructs and cloned into pRAB11 [10]. Primers used for cloning purposes are listed in Supplementary Table S1. The integrity of all constructs was verified by sequencing.

### Recombinant protein production

The recombinant EssC-N protein was obtained by cultivating freshly transformed *E. coli* BL21(DE3) pLysS in a 20 ml starter culture of Luria–Bertani (LB) medium including 50 μg/ml kanamycin and 15 μg/ml chloramphenicol. A 5 ml volume of starter culture was used to inoculate 1 litre of LB culture (total 4 litres) including 1 mM magnesium chloride and 0.5 mM calcium chloride and grown at 37°C in 5 litre Erlenmeyer flasks. When the *D*_600_ reached 0.6–0.8, expression was induced with 1 mM IPTG (isopropyl-β-D-thiogalactopyranoside) and the culture was incubated overnight at 25°C. The cells were harvested by centrifugation (5000 ***g***, 30 min, 4°C) then resuspended in buffer A (50 mM sodium phosphate, pH 7.8, 300 mM sodium chloride 10% glycerol) along with 1 mM DTT and a Mini Protease inhibitor tablet (Roche). The cell/buffer A mixture was processed in a French press (Thermo) and the lysed sample was passed through a 0.45 μm filter and supplemented with 25 mM imidazole ready for the first affinity chromatography purification step. A 1 ml CV (column volume) His-Trap HP column (GE Healthcare) charged with Ni^2+^ was calibrated by four CV washes with buffer B (50 mM sodium phosphate, pH 7.8, 25 mM imidazole, 300 mM sodium chloride and 10% glycerol) after which the sample was loaded and fractions were collected over a linear imidazole concentration gradient (25–250 mM over 18 CV). Fractions containing eluted EssC-N were combined and concentrated in a spin concentrator (Sartorius) exchanging the buffer with buffer A and the sample was incubated overnight with His-tagged TEV protease (molar ratio of 1:20 TEV/recombinant protein) at 4°C. The sample was passed through a His-Trap HP column previously equilibrated with buffer A, to separate the desired sample from non-cleaved protein and the TEV protease. Fractions containing EssC-N were concentrated and further purified using a Superdex75 GL10/300 size-exclusion chromatography (SEC) column equilibrated with buffer C {10 mM Tris/HCl, pH 7.8, 20 mM sodium chloride and 0.5 mM TCEP [tris-(2-carboxyethyl)phosphine hydrochloride]}. Fractions containing purified EssC-N were combined, concentrated to 20 mg/ml in different buffers and used in crystallization trials.

An SeMet-labelled form of EssC-N was obtained using a metabolic inhibition protocol [11]. For the starter culture freshly transformed *E. coli* BL21(DE3) pLysS cells were grown in 50 ml of LB broth along with 50 μg/ml kanamycin and 15 μg/ml chloramphenicol. The cells cultured for 14 h at 37°C. The starter culture was harvested, pelleted and washed with M9 medium and used to inoculate 2 litres of M9 medium. The *D*_600_ reached 0.6–0.8 after approximately 4 h and the culture was supplemented with an amino acid stock containing 60 mg/l L-selenomethionine, 100 mg/l L-phenylalanine, L-lysine and L-threonine, and 50 mg/l L-isoleucine, L-leucine and L-valine, then expression was induced by addition of IPTG and the culture was incubated for 3 h at 37°C. Purification of the SeMet-labelled EssC-N followed the same protocol described for the native form of the protein.

To obtain EssC-C, similar protocols to those adopted for production of EssC-N were used and following removal of the His-tag the sample was dialysed against buffer A. For ATP-binding and hydrolysis experiments, the final SEC step was carried out in buffer D (50 mM Tris/HCl, pH 7.8, 100 mM sodium chloride, 2 mM magnesium chloride and 0.5 mM TCEP). SeMet-substituted EssC-C was obtained using the same metabolic inhibition protocol outlined and the protein purified as for the native sample.

The level of purity, mass of the recombinant proteins and that full incorporation of SeMet had been achieved were determined by matrix-assisted laser-desorption ionization–time-of-flight mass spectrometry using an Applied Biosystems Voyager DE-STR spectrometer (University of Dundee ‘Fingerprints’ Proteomics Facility).

The preparation of full-length membrane-bound EssC followed the same protocol used for EssC-C except that *E. coli* LEMO21(DE3) cells were used, the culture medium contained 200 μM L-rhamnose and gene expression was induced with 400 μM IPTG [12]. Membranes were isolated by centrifugation (150000 ***g***, 60 min, 4°C), after cells were disrupted in a French press. The material was resuspended in buffer A and solubilized with *n*-dodecyl-β-D-maltoside (DDM) at a protein/detergent mass ratio of 1:3 for 2 h at 20°C. Non-solubilized material was removed by centrifugation and the resulting supernatant was treated essentially as described above using buffers supplemented with 0.03% (w/v) DDM and a Superdex 200 GL 30/100 column (GE Healthcare) for SEC. The yields of EssC-N, EssC-C and EssC were estimated on the basis of theoretical molar absorption coefficients at 280 nm of 26930, 41510 and 149950 M^−1^·cm^−1^ respectively, calculated using VectorNTI (Invitrogen). Both SEC columns used were calibrated with molecular mass standards (thyroglobulin, 670 kDa; γ-globulin, 158 kDa; serum albumin, 67 kDa; ovalbumin, 44 kDa; myoglobin, 17 kDa; vitamin B_12_, 1 kDa).

### *S. aureus* sample preparation and Western blotting

*S. aureus* strain RN6390 and the isogenic Δ*essC* deletion variant [3] were used for protein expression and secretion analysis. Cells were grown overnight in tryptic soy broth (TSB) at 37°C under vigorous agitation, diluted 1:100 into fresh TSB medium and growth was monitored by measuring the *D*_600_. Where necessary, chloramphenicol, to a final concentration of 10 μg/ml, was added for plasmid selection. Protein production was induced by addition of anhydrotetracycline when the *D*_600_ of the culture reached 0.4. Cells were grown until the culture reached a *D*_600_ of 2. Samples were withdrawn and the cells were separated from the culture supernatant by centrifugation at 2770 ***g***. Supernatant samples were passed through a 0.22 μm filter, directly mixed with an appropriate amount of 4× NuPage LDS loading buffer and boiled for 10 min. For whole cell fractions, pelleted cells were washed with 1× PBS and normalized to a *D*_600_ of 2 in 1× PBS. Cells were lysed by the addition of lysostaphin (Ambi) to a final concentration of 50 μg/ml and incubated at 37°C for 30 min. Samples were mixed with an equal volume of 2× LDS loading buffer and boiled for 10 min.

For the preparation of *S. aureus* membrane fractions, 10 ml of *D*_600_ 2 cells were pelleted and washed as described above and resuspended in 1 ml of 1× PBS plus Mini Protease inhibitor (Roche). Digestion of the cell wall was carried out using lysostaphin and cells were broken by sonication. Intact cells were removed by centrifugation (10000 ***g***, 10 min, 4°C) and membrane fractions were prepared by ultracentrifugation (227000 ***g***, 30 min, 4°C). Membrane pellets were solubilized in 1× PBS and 0.5% Triton X-100 and the supernatant was kept as the soluble protein fraction. Protein concentration of the membrane and soluble fractions was determined with the DC™ Protein Assay (Bio-Rad Laboratories). For preparation of urea-washed membrane fractions, broken cells were thoroughly mixed with an equal volume of 8 M urea prior to ultracentrifugation. Membrane pellets were washed with 1× PBS to remove residual urea and resuspended as described above.

For Western blots, samples were mixed with LDS loading buffer and boiled for 10 min prior to loading on Bis-Tris gels. Western blots were performed according to standard methods with antibody dilutions as follows: anti-EsxA 1:2500, anti-EsxC 1:2000, anti-EsaA 1:10000, anti-EssC 1:10000 and anti-TrxA 1:20000.

### Binding of mant-ATP

A 1 mM stock solution of the fluorescent mant-ATP (2′/3′-(*N*-methyl-anthraniloyl)-adenosine-5′-triphosphate triethylam-monium salt; Jena Bioscience) was prepared in buffer D and titrated with EssC-C. Fluorescence was monitored at 20°C using a LS44 spectrometer (PerkinElmer) with an excitation wavelength of 350 nm (or 360–550 nm for the emission spectrum) and an emission wavelength of 448 nm at a bandwidth of 5 nm. Bound mant-ATP was displaced by titration with 100 mM ATP.

### Blue native polyacrylamide gel electrophoresis of EssC

Linear 4–12% gradient native Bis-Tris gels (Novex®, Life Technologies) were used according to the manufacturer's protocol. Samples comprising 8 μg of purified EssC-C or 5 μg of full-length EssC were loaded. Apoferritin (480 kDa), B-phycoerythrin (242 kDa), lactate dehydrogenase (146 kDa), BSA (66 kDa) and soyabean trypsin inhibitor (20 kDa) were used as standard proteins. The EssC band was excised from the BN-PAGE (blue native polyacrylamide gel electrophoresis) gel, digested with trypsin and subjected to peptide mass fingerprinting using matrix-assisted laser-desorption ionization–time-of-flight mass spectrometry analysis (University of Dundee ‘Fingerprints’ Proteomics Facility).

### Crystallographic analyses

Native EssC-N crystals, maximum dimension 0.2 mm, were obtained in 2 days by sitting-drop vapour diffusion at 20°C in 1 μl drops at the ratio of 1:1 protein solution (20 mg/ml EssC-N in 50 mM sodium phosphate, pH 7.8, 300 mM sodium chloride and 0.5 mM TCEP) to reservoir condition (0.2 M magnesium formate and 20% w/v PEG3350). Isomorphous SeMet-labelled EssC-N crystals were grown in the same fashion but with the protein concentration halved. Crystals were harvested using glycerol or a mineral oil cryo-protectant (LV-oil, MiTeGen), flash frozen in liquid nitrogen and characterized in-house with a Rigaku MicroMax 007HF generator equipped with VariMax VHF optic, and a Saturn944 HG+ charge-coupled device (CCD) detector. Data were then collected, at −173°C, on beamline I04 at the Diamond Light Source (DLS, Didcot, U.K.) with a Dectris Pilatus 6M-F detector. The data were indexed and merged using XDS [13] and SCALA [14] respectively. The crystals displayed the space group *P*2_1_2_1_2_1_. The structure could not be determined using the native data; however, the SeMet-labelled crystal structure was solved to 2.1 Å (1 Å=0.1 nm) using the SAD protocol of Auto-Rickshaw [15]. A partial model of 63 residues was produced during automated building using the program ARP_wARP [16]. *F*_A_ values (the non-anomalous component of the Bijvoet pair) were calculated using SHELXC [17]. The maximum resolution for substructure determination and initial phase calculation was set to 2.5 Å. Three out of four selenium atoms were found using SHELXD [18]. Initial phases produced a CC (correlation coefficient) of 0.29. The occupancy of all substructure atoms was refined using the program BP3 [19]. Density modification, and phase extension were performed using RESOLVE [20]. The CC increased to 0.74 at 2.1 Å resolution and the model was extended to 157 residues with BUCCANEER [21], as implemented in the CCP4 suite of programs [22]. This resulted in *R*_work_ and *R*_free_ of 0.25 and 0.31 respectively. This model was inspected, along with electron density and difference density maps, adjusted and extended to 184 residues using COOT [23] and then refined with Refmac5 [24]. Translation/Libration/Screw (TLS) refinement [25] in Refmac5 with multiple rounds of electron and difference density map inspection, model manipulation and the inclusion of water molecules and dual conformers completed the refinement.

Crystals of SeMet EssC-C, and the native complex with mant-ATP were grown under oil at 20°C. A volume of 1 μl of protein solution (15 mg/ml EssC-C in 10 mM sodium phosphate, pH 7.8, 20 mM NaCl, 10% glycerol, 0.5 mM TCEP and 2 mM cysteine) was mixed with 1 μl of precipitant solution [0.1 M sodium cacodylate, pH 6.3, 200 mM calcium acetate, 18% (w/v) PEG300] in a Terasaki Microbatch plate (Molecular Dimensions) and covered with silicone oil (Molecular Dimensions). For co-crystallization, 2 mM mant-ATP and magnesium chloride were added to the protein solution. Isomorphous orthorhombic crystals formed after 2–3 weeks and grew up to 0.8 mm in length over a further 2 weeks. The crystals were picked up in LV-oil (MiTeGen), cooled in a stream of gaseous nitrogen at −173°C then characterized in-house and diffraction quality was assessed. X-ray data from SeMet-labelled EssC-C were collected on BM30A at the European Synchrotron Radiation Facility (ESRF, Grenoble, France) and data of nucleotide analogue-bound native EssC-C at beamline I04 at Diamond Light Source, in each case with an ADSC Q315r CCD detector. Data were processed using XDS and SCALA. The crystals display space group *I*222 with two polypeptides (labelled A and B, total 1034 residues) in the asymmetric unit.

The EssC-C crystal structure was, as for EssC-N, solved using the SAD protocol of Auto-Rickshaw. Analysis of the data suggested that 3.5 Å was the appropriate resolution for substructure determination and initial phase calculation. Subsequently 31 out of 32 selenium atom positions were identified and occupancy refined. Initial phases produced a CC of 0.38. Density modification, phase extension and two-fold non-crystallographic symmetry (NCS) averaging were performed with RESOLVE. A partial model of 501 residues was made with HELICAP [26]. Model phases were combined with the experimental phases and a further round of density modification gave a CC of 0.63 at 2.8 Å. The model was extended to 947 residues with BUCCANEER as implemented in the CCP4 suite. This resulted in *R*_work_ and *R*_free_ values of 0.37 and 0.46 respectively. Inspection of electron and difference density maps combined with model building using COOT extended the model to 1004 residues. Refinement with Refmac5, and employing strict NCS restraints followed and reduced *R*_work_ and *R*_free_ values to 0.33 and 0.44 respectively. However, the N-terminal region, the D2–D3 linker region and the interface where intermolecular symmetry contacts occur were poorly defined.

Improved diffraction data were obtained from an isomorphous crystal of the native EssC-C–mant-ATP complex. The partial model of the SeMet protein was used for molecular replacement with PHASER [27]. Two-fold map averaging using PARROT [28] followed by model building using BUCCANEER generated a model of 991 residues, which gave *R*_work_ and *R*_free_ values of 0.30 and 0.37. TLS refinement in Refmac5 with iterative rounds of map inspection, model manipulation and the inclusion of nucleotide ligands, water molecules, ethylene glycol and several side-chain conformers completed the refinement. In one molecule there was good density for the mant moiety of the ATP derivative, in the other molecule the density for this part of the nucleotide ligand was ambiguous and so ATP was modelled. In the early stages of the analysis we included a Mg^2+^ ion positioned near the ATP phosphates but it did not refine satisfactorily and so was then removed. NCS restraints were applied at a domain level and maintained throughout the refinement.

Crystallographic statistics are presented in Supplementary Table S2. MOLPROBITY [29] and COOT were used to monitor model geometry during refinement of both EssC-N and EssC-C. Figures were prepared using PyMOL (Schrödinger LLC). The DALI server was used to search the PDB for structural homologues and structural superpositions were performed using DALILITE [30]. Multiple sequence alignments were calculated using ClustalW2 [31] and edited using ALINE [32]. Crystal contact interfaces were analysed using PISA [33].

### Computational modelling

The EssC transmembrane segments were predicted using TMHMM v2.0 [34]. Homology models were generated using the one-to-one threading mode of the Phyre2 fold recognition server [35]. A homology model of EssC-D1 (Ala^643^–Ile^898^) was made on the basis of 231 residues in D2 and 278 residues in the ATPase domain of the hexameric FtsK (PDB code 2IUU [36]), and superimposed on the latter.

## RESULTS AND DISCUSSION

### Characterization of EssC and EssC-C

Recombinant EssC-C, the C-terminal residues 966–1479 comprising the two ATPase-like modules D2 and D3 (Figure 1), was produced in *E. coli* and purified with a yield of 7 mg/l culture. The protein, approximately 60 kDa, migrates in SEC and BN-PAGE with apparent molecular masses of approximately 83 kDa and 65 kDa respectively, which is consistent with a monomeric state. An additional higher-molecular-mass band (180 kDa), accounting for approximately 5% of the total protein, was observed in the BN-PAGE analysis suggestive of some aggregation (Supplementary Figure S1).

An equilibrium binding constant of 0.94±0.11 μM for the ATP analogue mant-ATP with EssC-C was determined on the basis of fluorescence measurements. The binding curve suggests a stoichiometry of <1:1 (mant-ATP/EssC-C) as an excess of protein was required to reach saturation. The mant-ATP fluorescence observed when bound to EssC-C was quenched by addition of ATP and the analogue was readily displaced (Supplementary Figure S2).

Recombinant full-length 169 kDa EssC was detected in the *E. coli* membrane fraction, solubilized and purified with yields of typically approximately 10 μg/l bacterial culture. The purified material migrated in a BN-PAGE experiment at an apparent molecular mass of 1 MDa and the identity was confirmed by mass fingerprinting analysis (Supplementary Figure S3). The observed mass is consistent with the presence of a hexamer. Note also that a multimeric form of EssC with a similar high molecular mass was detected from *S. aureus* extracts in a recent cross-linking study [37].

### Structure of EssC-N

**Figure 2:**
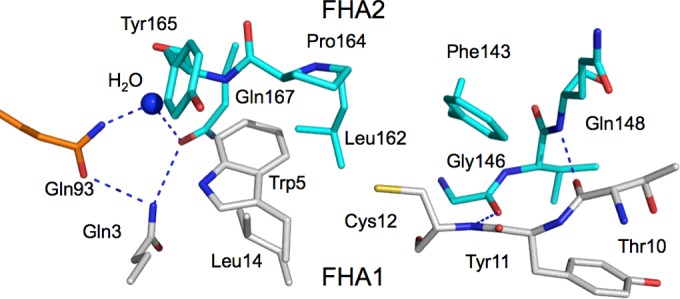
Residues that contribute to the FHA1–FHA2 interface The residues that contribute to the inter-domain interface are depicted in stick mode coloured according to atom type (N blue, O red, S yellow) with grey C positions corresponding to FHA1, cyan to FHA2 and orange for Gln^93^ that resides on the inter-domain linker. A water molecule is shown as a blue sphere and potential hydrogen bonds as blue dashed lines.

The EssC-N crystals, although of good appearance, displayed diffraction that indicated the presence of multiple crystalline components. It proved surprisingly difficult to determine the structure, and numerous samples were tested before useable data were recorded. Initial phases were derived from a SAD experiment using SeMet-substituted protein and it was this model that was subsequently refined. EssC-N consists of residues Met^1^–Leu^195^ folded into a two-domain structure with each domain representative of the β-sandwich FHA fold (Figure 1). Residues Met^1^–Glu^91^ constitute domain FHA1, which is linked to FHA2 via a short dipeptide linker (Glu^92^ and Gln^93^). Seven residues are missing from the model of FHA1 due to insufficient electron density. These residues (at positions 19, 20, 53, 54, 80, 81 and 82) are located on turns linking β-strands. FHA1 and FHA2 domains consist of nine and 12 β-strands respectively with the domains aligned almost perpendicular to each other. The domain–domain contacts are mainly via van der Waals interactions between side chains. These interactions involve residues on β1, β2, β8, β9 and the turns that link β1–β2 and β7–β8 on FHA1, with the turns linking β15–β16, β17–β18 and β19–β20 on FHA2. A pronounced feature at the domain–domain interface is Trp^5^ nestled into a cavity formed by Leu^162^, Pro^164^ and Tyr^165^ (Figure 2). There are van der Waals interactions formed between Cys^12^ and Leu^14^ of FHA1 with Phe^143^ and Leu^162^ respectively on FHA2. Hydrogen-bonding interactions also contribute to the domain alignments, with a network of three glutamines (residues 3, 93, and 167) and a water molecule interacting at one end of the interface. In addition, Gln^93^ lies on the inter-domain linker section and interacts with glutamines on each FHA domain, whereas at the other end of the interface two hydrogen bonds involving main-chain amide and carbonyl group link Thr^10^ and Tyr^11^ with Gly^146^ and Gln^148^ respectively to form a short section of antiparallel β-sheet (Figure 2).

The most similar structural orthologue identified is also a twin-FHA domain structure, namely the *S. aureus* EssC N-terminal domains (PDB code 1WV3 [7]), which shares 15% sequence identity. Superimposition of the twin domains, comprising some 172 Cα positions, results in an rmsd of 2.9 Å (*Z*-score 18) and shows the alignment of the FHA domains with respect to each other is conserved in the two structures (Supplementary Figure S4). When individual domains are considered the rmsd falls to 1.7 Å (*Z*-score 10.5) over 71 Cα positions and an rmsd of 1.9 Å (*Z*-score 14.0) over 97 Cα positions for FHA1 and FHA2 respectively. Although the identity of amino acids is poorly conserved at the interface in general, the hydrophobic nature of the side chains is maintained and a series of compensating changes result in the same overall structure. For example, the Trp^5^:Leu^162^–Pro^164^–Tyr^165^ configuration in *G. thermodenitrificans* Essc-N is replaced by Ile^5^–Leu^12^–Tyr^77^–Gln^75^:Try^158^–Gly^161^ in the *S. aureus* protein structure. The main differences between the structures are that the FHA1 β6–turn–β7 segment of EssC-N is truncated by eight residues and in FHA2, the β13–turn–β14 section extended by five residues.

EssC-N also shares structural similarity with proteins involved in phosphopeptide binding: at the level of single FHA domains then alignments result in rmsd values of 1.5 Å (*Z*-score 9.7, 76 Cα positions) for FHA2 and 2.5 Å (*Z*-score 8.2, 78 Cα positions) for FHA1 with the NMR-derived structure of Ki67 (PDB code 2AFF [38]).

The FHA domain was first identified in forkhead transcription factors and subsequently in numerous proteins from both prokaryotes and eukaryotes [39]. The proteins in which they are found are implicated in diverse biological events including signal transduction, transcriptional regulation and vesicular transport, although the precise contribution of the FHA domains to these processes remains elusive. In some examples the domain can bind phosphothreonine-containing peptides so providing a mechanism to link phosphorylation with direct protein–protein interactions [39]. However, as reported by Tanaka et al. [7], motifs common to many FHA domains and residues implicated in the recognition and binding of phosphothreonine, as exemplified by Ki67 are absent from EssC-N.

We made numerous attempts, all unsuccessful, to identify protein binding partners for the FHA domains using pull-down and bacterial two-hybrid approaches (results not shown). The only functional contribution we can assign to this EssC segment at present is in making a contribution to producing a stable, active protein (see below). It is intriguing, however, that the orientation of the FHA domains with respect to each other is conserved in the two structures discussed, in spite of a low sequence identify, and this suggests a functional role for such a structural combination.

### Structure of EssC-C

The crystal structure of EssC-C was phased from a SAD experiment using an SeMet-substituted protein. A crystal of the EssC-C–mant-ATP complex gave improved data and hence this dataset was used to finalize the structure at 2.9 Å resolution (Figure 1). The asymmetric unit consists of two EssC-C polypeptides, labelled A and B, each resolved from Ala^968^ to the C-terminal Glu^1479^. The polypeptide is composed of two homologous P-loop ATPase-like modules or domains (D2 and D3), which are connected by a linker (Gln^1225^–Ser^1241^). This domain structure is dominated by a central β-sheet core of seven or eight strands mainly aligned in parallel fashion and decorated with helices on either side. In D2 the central parallel β-sheet is sandwiched between α1–α2 and α3–α6 and in D3 between α10–α12 and α13–α17. In D3 the core is extended with four strands β11, β12, β19 and β20. Strand β19 follows a sharp turn in the vicinity of the potential ATP-binding site. In D2 the corresponding polypeptide does not extend the core sheet but rather forms an extended structure which we term the ‘β9-segment’ (Ile^1171^–Tyr^1198^) that protrudes from the globular fold so that β9 (Gly^1183^–Lys^1188^) serves to extend the central β-sheet of a symmetry-related molecule D2 domain through association with β8 (Thr^1155^–His^1159^) and β10 (Thr^1195^–Ile^1197^) (Supplementary Figure S5). A least-squares fit of polypeptide A and B, over 457 Cα-atoms, results in an rmsd of 2.1 Å. This relatively high value results from the different positioning of the two domains with respect to each other, which is facilitated by different conformations of the linker region. Aligning individual domains for molecules A and B results in an rmsd of 1.2 Å for D2 (231 Cα-atoms) and 0.6 Å for D3 (211 Cα-atoms).

### The D2 domain

The initial structure of SeMet–EssC-C indicated that ATP was present in the nucleotide-binding site of the D2 module even though nucleotide had not been added during the crystallization. The structure derived from crystals grown in the presence of mant-ATP displayed additional density for the mant moiety in one polypeptide (Supplementary Figure S6). ATP was included in the other since there was no clear density for this mant moiety. Aspects of protein–nucleotide recognition in EssC-C are similar to the related Type IV pilus retraction (PilT) ATPase [40,41]. The nucleotide binds in a polar cleft created on one side of the β-sheet by several segments of the polypeptide fold. Both Walker A and B type motifs [42] are evident (Supplementary Figure S7). The β3–α1 loop encompasses the Walker A motif and also the N-terminal dipole of this helix, the β4–α2 and β10–α9 segments and the C-terminal part of β6 carries the Walker B motif. The Walker A motif lysine [43], Lys^1007^, and the backbone amides of Gly^1004^, Gly^1006^, Lys^1007^ and Thr^1009^ donate hydrogen bonds to α- and β-phosphates (Supplementary Figure S6). The side chains of Lys^1007^ and Thr^1009^ form hydrogen bonds with β- and α-phosphate groups respectively. The γ-phosphate participates in solvent-mediated hydrogen bonds to the protein and direct interactions with side chains of Ser^1008^, Asn^1036^ and Asp^1105^. Asp^1105^, in the Walker B motif, is probably protonated to allow this association. The adjacent residue in EssC-C D2 is Asn^1106^ rather than the canonical acidic residue that primes a water molecule for nucleophilic attack on the γ-phosphate [44]. This is unusual and, to the best of our knowledge, no P-loop ATPase with such a variation of the Walker B motif has been described. Indeed a similar substitution of glutamine for the Walker B glutamate has been shown to abolish ATPase activity but not ATP binding [45,46].

The adenine N6 donates a hydrogen bond to the main-chain carbonyl of Ile^1197^, whereas N1 interacts with Gln^1196^ via a water molecule. In similar fashion, N7 is linked to Thr^1010^ OG1 and the Gly^1006^ carbonyl by another water molecule. Asp^1207^ holds the ribose in place by a hydrogen bond with the O2’ hydroxy group. The mant group is accommodated in a hydrophobic pocket formed by Val^1209^ and Leu^1210^ on α9 and Ala^1038^ on α2 (Supplementary Figure S6).

**Figure 3:**
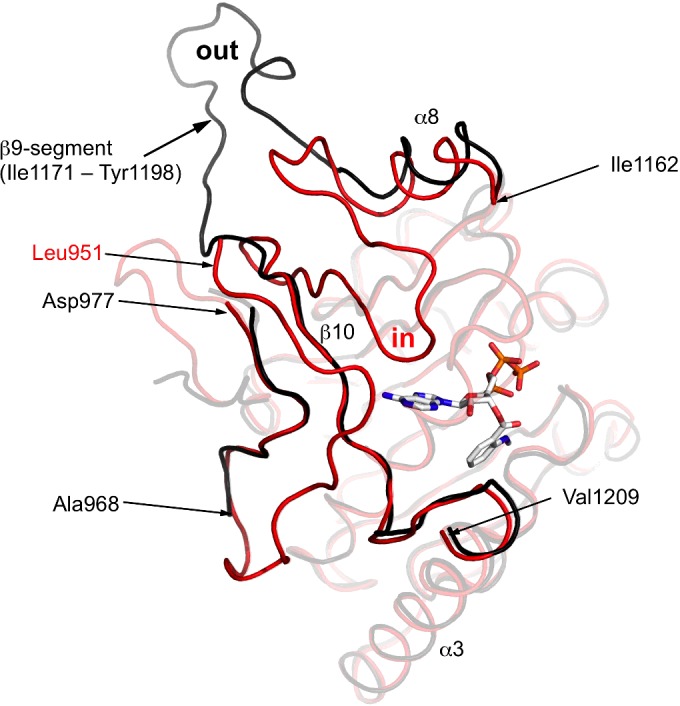
A comparison of the EssC-C D2 ‘in’ and ‘out’ conformations The least-squares overlay of D2 (black ribbon) with the equivalent domain of PDB code 4LYA (red ribbon). The mant-ATP is shown in stick mode coloured according to atom type as in Figure 1. Non-transparent regions highlight the different positioning of the β9-segment (labelled ‘in’ and ‘out’) and the extended N-terminus of PDB code 4LYA*.*

A comparison of D2 with the structure of the slightly longer construct derived from the same protein (PDB code 4LYA [8]), independently determined, shows that the structures, although very similar, display a significant difference. In our structure the segment between residues Ile^1171^–Tyr^1198^ extends away from the core of the domain to form what we term the β9 segment in an ‘out’ conformation. In PDB code 4LYA, this segment is tucked back on the core structure forming two β-strands and a tight turn between them, the ‘in’ conformation (Figure 3). The first strand is positioned beside and interacts with the β5–α3 segment, whereas the second strand serves to extend the core β-sheet interacting with β5 on one side then β10 on the other. The turn is placed near the adenine-ribose components of the nucleotide ligand. The construct used to generate PDB code 4LYA starts at residue 951, whereas ours is shorter and starts at 961. The extended N-terminal structure in PDB code 4LYA forms an edge of the adenine-binding pocket, and participates in a number of interactions with the polypeptide that adopts the ‘in’ conformation. The presence of the ‘in’ conformation for the β9 segment and the N-terminal extension are, however, not essential requirements for nucleotide binding since ATP or a derivative was present in the ‘out’ conformation structure as well. Nevertheless, the striking conformational adaptability shown by the β9 segment hints at the possible structural changes that might occur as EssC supports a motor transport process.

### The D3 domain

Domains D2 and D3 share a sequence identity of 16% and structural alignments within molecules A and B, of 194 and 179 Cα-atoms results in rmsd values of 2.8 and 2.6 Å respectively. Major differences are that helices α4 and α8 of D2 are absent from D3. The latter contributes to distinct polypeptide conformations for the β9 segment, which extend the core sheet by contributing β9 and β19 respectively. The β9 segment of D2 then participates in inter-molecular contacts as described above. The corresponding region of the polypeptide in D3 is folded over the central β-sheet adopting a conformation commonly observed in P-loop-containing NTPases.

**Figure 4:**
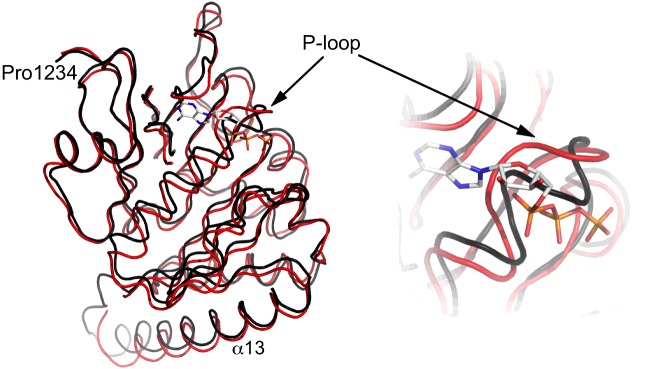
A comparison of the P-loop conformations in D3 structures Left: superimposition of EssC-C D3 (black ribbon) and the equivalent domain of PDB 4LYA (red ribbon). The bound ATP from PDB code 4LYA is depicted is similar fashion to the nucleotide in Figure 1*.* Right: a magnified view of the nucleotide-binding site shows the different conformations of the P-loop.

In our structure the D3 domain appears incapable of ATP binding. Despite the inclusion of excess mant-ATP in the crystallization conditions we only observe the ligand to bind the D2 domain and this is consistent with binding data that suggest a stoichiometry <1. However, Rosenberg et al. [8] report a structure (PDB 4LYA) in which both D2 and D3 ATP-binding sites are occupied. A comparison of the structures indicates that conformational differences in the D3 P-loop produce a helical structure (α10), which occludes the ATP-binding site (Figure 4). The reasons for this difference are unclear, but comparisons clearly indicate conformational pliability in this ligand-binding site.

The Walker A motif is clearly recognizable in D3 (Gly^1284^–Thr^1291^) (Supplementary Figure S7). However, in some EssC orthologues, e.g. *Staphylococcus* spp., the third domain lacks ATP-binding motifs entirely, even though homology modelling predicts a similar fold. Although EssC from *G. thermodenitrificans* and *S. aureus* share an overall sequence identity of approximately 32%, the similarities vary depending on specific domains, amounting to 65, 32 and 13% when comparing the ATPase modules D1, D2 and D3 respectively. Very recently a genomic survey revealed that there is unexpected diversity in *S. aureus* EssC [47]. The EssC sequences were shown to fall into four different groupings, with the sequence diversity largely confined to the D3 module. Intriguingly, each of the four EssC variants was associated with different candidate suites of secreted substrates, implicating the D3 domain in substrate selectivity [47].

Site-directed mutagenesis to disable Walker A and B motifs contributions to ATPase and nucleotide-binding activity in the three domains of the *B. subtilis* EssC orthologue, called YukBA, revealed that only the enzyme activity of D1 is essential for secretion [48]. We speculate that D1 presents a well-conserved ATPase activity that is essential for the mechanics of translocation but that D2 and in particular D3 may have evolved to specialize by interaction with particular cargoes for the distinct secretion systems.

### The D3 domain is essential for Type VII secretion

**Figure 5:**
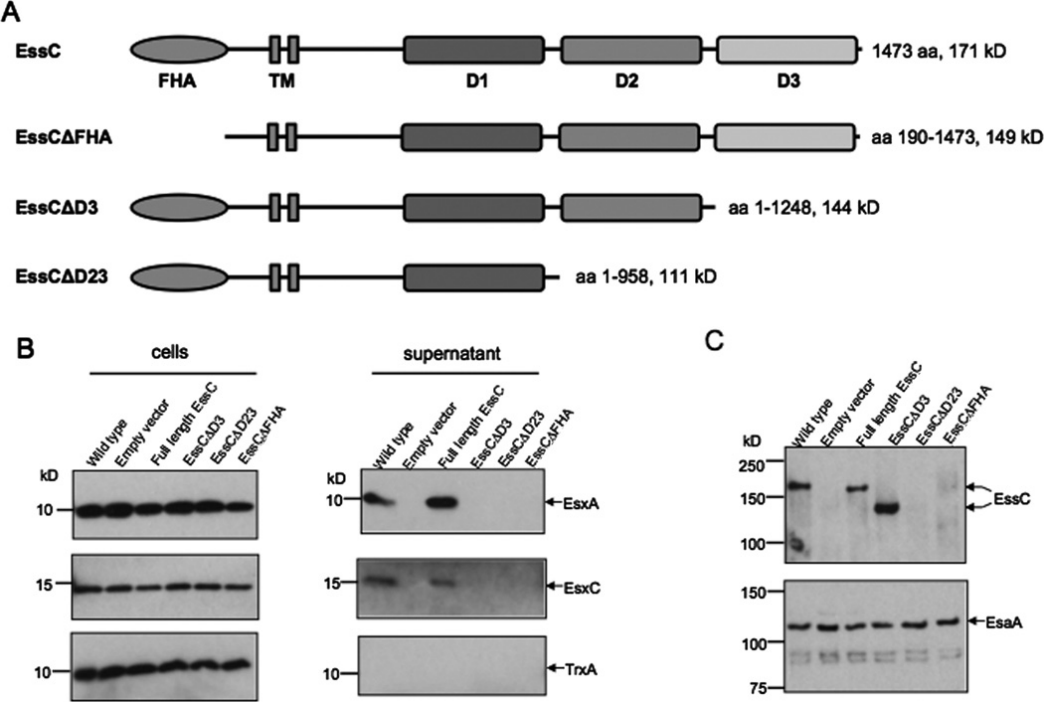
Truncation analysis of EssC (**A**) Schematic representation of the *S. aureus* EssC truncations constructed during the present study. (**B**) Whole cell (left panels) or supernatant (right panels) samples were prepared from *S. aureus* strain RN6390 (wild-type) or the isogenic *essC* deletion strain harbouring pRMC2 alone (empty vector) or encoding the indicated EssC variants. Strains were cultured as described in the Materials and methods section, and expression from pRMC2 derivatives was induced by addition of 25 ng/ml anhydrotetracycline. For each sample 11 μl of culture supernatant and 4 μl (EsxA) or 10 μl (EsxC, TrxA) of cells adjusted to a *D*_600_ of 1 were loaded. Samples were separated on 15% Bis-Tris gels and immunoblotted with antibodies raised against the Ess secreted substrates EsxA, EsxC or the cytoplasmic control TrxA. (**C**) Volumes of 10 ml of cells from the same samples in (**B**) were adjusted to a *D*_600_ of 1, separated on 8% Bis-Tris gels and blotted with an anti-EssC antibody (top panel). The same samples were probed with an anti-EsaA antibody as a loading control (bottom panel).

Informed by our structural models we carried out a truncation analysis to determine which EssC domains might be essential for T7SS function in a tractable model system. We designed truncations to produce *S. aureus* EssC lacking ATPase D3 alone (EssCΔD3), D2 and D3 (EssCΔD23) or the N-terminal FHA domains (EssCΔFHA; Figure 5). Removal of the most C-terminal ATPase module, D3, alone was sufficient to completely abolish secretion of either EsxA or EsxC substrates, without affecting the apparent stability or membrane integration of EssC (Figure 5 and Supplementary Figure S8). The deletion of D2 and D3 also abolished substrate secretion, although in this case truncated EssC was not detected in the membrane (Supplementary Figure S9). Note that, it was necessary to provide this variant with a C-terminal HA tag since it lacks the epitope that is recognized by the EssC antiserum. Removal of the *S. aureus* EssC FHA domains also resulted in no detectable secretion of EsxA or EsxC but again no EssC variant could be detected by Western blotting. We conclude that the extreme C-terminal ATPase domain is essential for EssC activity possibly by interaction with cargo proteins and that the presence of D2 and FHA domains are required for the production of a stable protein.

Our findings contrast with a previous report that transposon insertions at either codon 1279, which is close to the start of encoding D3, or codon 721, which is part way through the sequence encoding D1, did not significantly affect secretion of EsxA or EsxB [2]. However, we could not rescue substrate secretion even if we induced expression of the EssC truncates with a high level of anhydrotetracycline (Supplemental Figure S8), although overproduction of non-truncated EssC did enhance substrate secretion suggesting that the amount of active EssC in wild-type cells may be a limiting factor for activity of the secretion system. Rosenberg et al. [8] suggest that the important contribution that D3 makes to the T7SS is in binding directly to a substrate, EsxB, and that this enforces a multimerization to form the functional secretion apparatus. Although an attractive model, again, our data contradict this in that full-length EssC from *S. aureus* [37] and *G. thermodenitrificans* oligomerize in the absence of EsxB. The data available suggest a complexity in the function of EssC that is not yet fully understood.

### Comparison with FtsK-ATPase and the generation of a hexameric model

**Figure 6:**
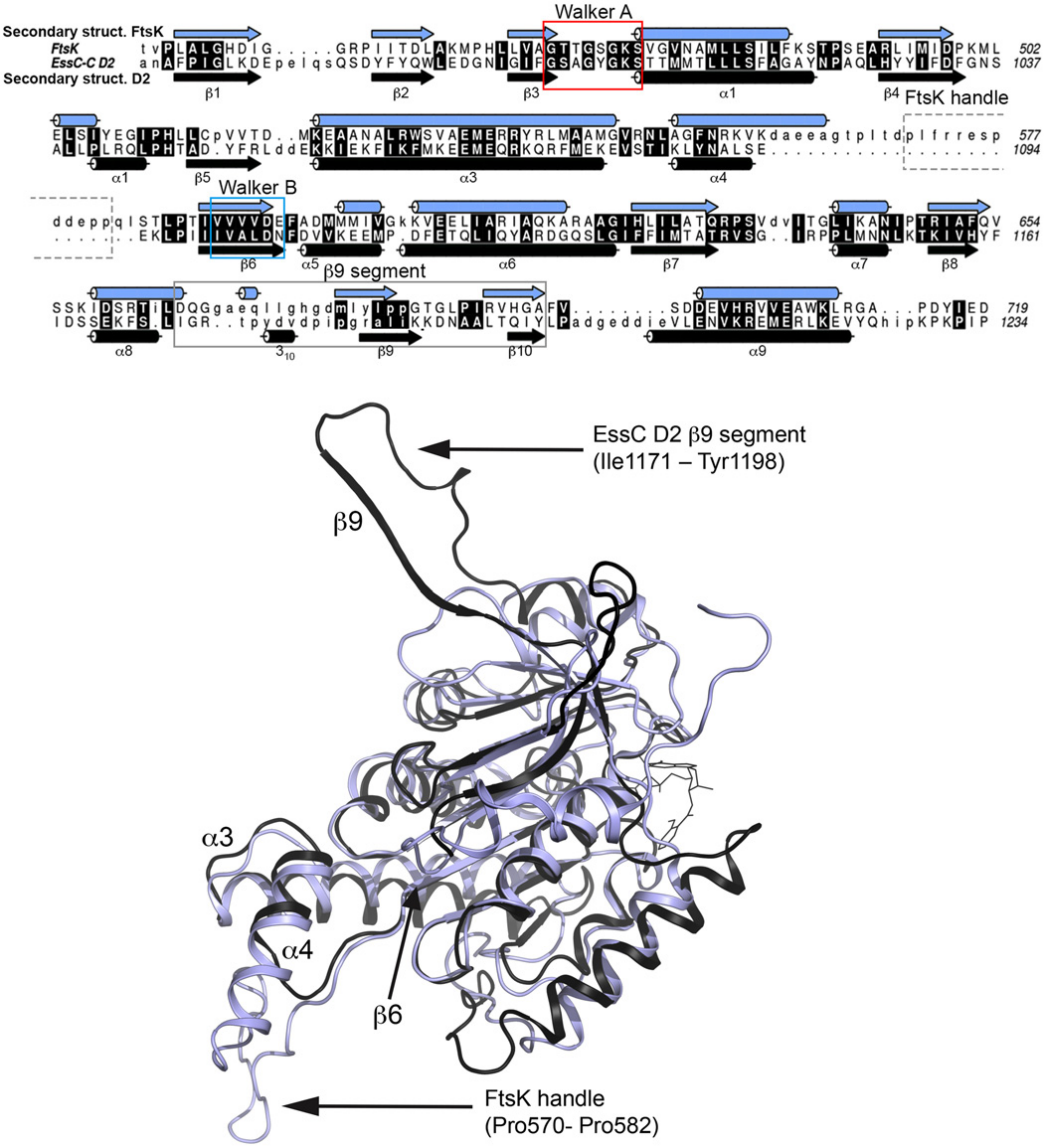
Sequence and structural alignment of EssC-C D2 and FtsK (**A**) Amino acid sequence alignment with assigned elements of secondary structure. The FtsK handle (dashed box), the D2 β9-segment (grey box) and signature motifs, as described in [53], are marked. The Walker A motif (red box), the invariant sequence pattern [A/G]XXXXGK[S/T], forms the P-loop that interacts with phosphate groups of the nucleotide and the Walker B motif (blue box) residues (hhhhDE with h=hydrophobic residue). (**B**) Ribbon depiction showing the superimposition of EssC-C D2 (black) and the ATPase domain of *P. aeruginosa* FtsK (blue, PDB code 2IUT [36]). The bound nucleotide analogue mant-ATP is drawn as sticks.

The conserved fold of the EssC-C D2 and D3 domains clusters with the PilT class of ATPases [40], in particular FtsK. A structural alignment of molecule A D2 with monomeric *Pseudomonas aeruginosa* FtsK (PDB code 2IUT [36]) gives an rmsd of 2.2 Å over 220 Cα-atoms and a *Z*-score of 22 with 20% sequence identity (Figure 6). Molecule A domain D3 aligns with an rmsd of 2.8 Å for 220 Cα-atoms, a *Z*-score of 20, again with 20% sequence identity. Notably, sequence comparison of domain EssC D1 with FtsK reveals a significantly higher sequence identity of 31%. Although the core of these structures overlay well, the β9-segment feature of D2 is absent from monomeric and hexameric forms of FtsK (PDB code 2IUU [36]). Rather, in this part of the ATPase domain it is D3 which matches most closely to FtsK. This is likely to be a result of the D2 ‘out’ conformation described above. A significant difference is the presence of an extended structure, or handle, in FtsK between α11 and β9, which corresponds to the positions of the EssC-C D2 α4–β6 and D3 α13–β16 loops. The FtsK handle (Pro^570^–Pro^580^) protrudes almost 20 Å from the C-terminal surface of the structure. In hexameric FtsK this extension mediates contacts between subunits and helps to create a stable dodecameric double ring assembly [36].

**Figure 7:**
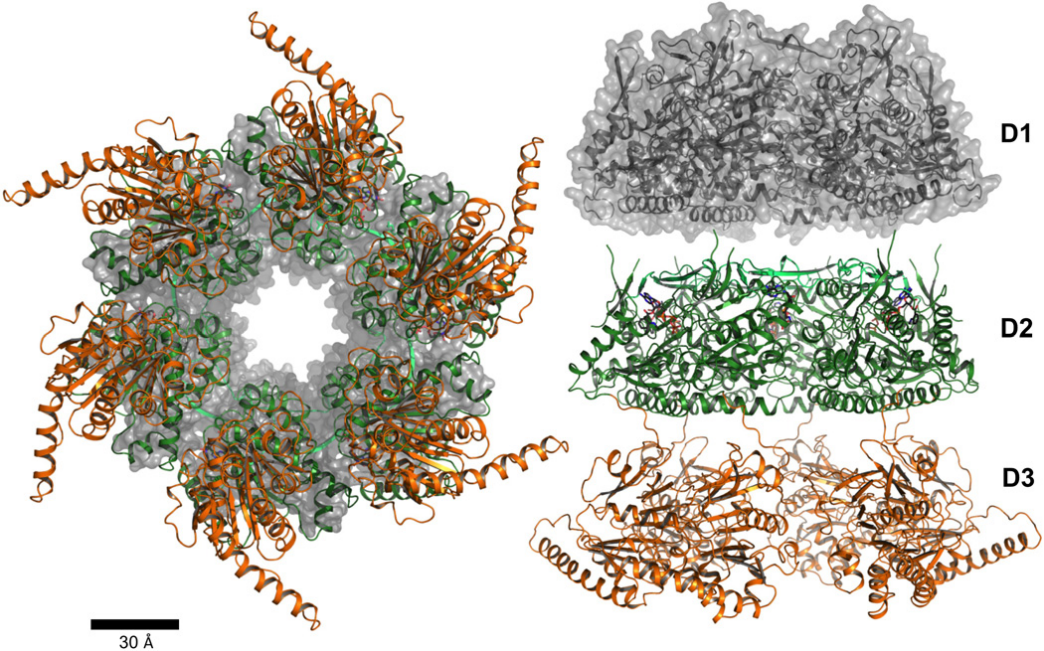
Hexameric model of the three ATPase domains The hexameric EssC-C model, with D2 (green) and D3 (orange), was generated by superimposition of D2 and the ATPase domain of hexameric FtsK (PDB code 2IUU [36]). EssC-C is drawn in cartoon representation with the bound nucleotide shown as sticks. A homology model of D1 is depicted as a grey semi-transparent van der Waals surface representation. The tube-like structure, formed by alignment of the three hexameric rings, is shown in side view (right).

A model of a multimeric EssC-C assembly was constructed by rigid body superimposition of D2 with hexameric FtsK and no further manipulation (Figure 7). This model does not show any steric clash for the position of the D3 domains, either with other D3 or adjacent D2 domains. The resulting pore with an inner diameter of approximately 30 Å would accommodate the ESAT-6 family protein dimer, which is the prototypical T7SS substrate [1]. Notably, the ESAT-6 family member EsxA from *G. thermodenitrificans* (PDB code 3ZBH), like the homologue from *S. aureus* [49] is a cylindrical structure displaying a polar, primarily acidic, surface.

## Conclusions

EssC and orthologues represent the only conserved membrane-bound component of the T7SS. This protein therefore represents a strong candidate as a component of the pore through which cargo is transported. EssC possesses ATPase-like domains as found in other proteins involved in transport processes and our data suggest that, in similar fashion, the full-length EssC forms a hexameric species. On the basis of comparison with the hexameric FtsK, we have generated a model which we propose represents the cytosolic entrance to the T7SS pore that would then be completed by the transmembrane helices.

Recent studies on the T7SS in the related *Mycobacterium marinum* and *Mycobacterium bovis* confirmed the critical role of the EssC homologue EccC_5_, in formation of an ESX-5 membrane complex that is functional for secretion [6]. Furthermore, on the basis of the observation that the middle and C-terminal domains of EccC_5_, equivalent to the D2 and D3 that we have studied, are affected by limited proteolysis, it was concluded that these domains are accessible and distant from the membrane. These observations match our model and this part of EssC, perhaps together with the FHA domains, might represent the hub for binding other T7SS components, including substrates. For *S. aureus* EssC it has been demonstrated that the first P-loop-containing domain, D1, alone can provide a motive force for secretion of EsxA and EsxB [2]. The remaining two domains may then play an auxiliary or regulatory role. This is reminiscent of VirB4, a soluble ATPase component of the Type IV secretion system, which also displays similarities to the FtsK/SpoIII-type family. VirB4 binds at the side of the Type IV core complex and does not interact with the DNA cargo directly [50].

The intriguing structural similarity of EssC to DNA-transporting ATPases, together with reports on the relevance of T7SSs in DNA transfer [51,52], highlights the possibility that the T7SS, like the Type IV secretion system, is a versatile secretion system that might be involved in transport of protein and or DNA. Future studies will address this important issue.

## ACCESSION NUMBERS

The co-ordinates and structure factor data for EssC-N and EssC-C have been deposited in the PDB under the accession codes 5FWH and 5FV0 respectively.

## Abbreviations

BN-PAGE: blue native polyacrylamide gel electrophoresis
CC: correlation coefficient
CCD: charge-coupled device
CV: column volume
DDM: *n*-dodecyl-β-D-maltoside
ESAT-6: early secreted antigenic target of 6 kDa
Ess: ESX-1 secretion system
FHA: forkhead-associated
FtsK: filamenting temperature-sensitive mutant K
HA: haemagglutinin
IPTG: isopropyl-β-D-thiogalactopyranoside
mant: 2′/3′-(*N*-methyl-anthraniloyl)
NCS: non-crystallographic symmetry
SAD: single-wavelength anomalous diffraction
SEC: size-exclusion chromatography
SeMet: selenomethionine
TSB: tryptic soy broth
T7SS: Type VII secretion system
TCEP: tris-(2-carboxyethyl)phosphine hydrochloride
TEV: tobacco etch virus
TLS: Translation/Libration/Screw
